# Multifactorial Evaluation of Atenolol, Caffeine, Carbamazepine and Ibuprofen on *Raphidocelis subcapitata* and *Chlorella vulgaris*

**DOI:** 10.3390/biology10090926

**Published:** 2021-09-16

**Authors:** Zaniel S. D. Procopio, Joanne B. Roberts, Colin Hunter, Ole Pahl

**Affiliations:** School of Computing, Engineering and Built Environment, Civil Engineering & Environmental Technology, Glasgow Caledonian University, 70 Cowcaddens Road, Glasgow G4 0BA, UK; zaniel.procopio@gcu.ac.uk (Z.S.D.P.); Joanne.Roberts@gcu.ac.uk (J.B.R.); O.Pahl@gcu.ac.uk (O.P.)

**Keywords:** algae, *Raphidocelis subcapitata*, *Chlorella vulgaris*, pharmaceuticals, micropollutants, ecotoxicity, multifactorial approach

## Abstract

**Simple Summary:**

This study analysed combined stress effects caused by micropollutants commonly found in the aquatic environment at extremely low concentrations. For this purpose, two microalgae were used as biomarkers and three characteristics of these organisms were combined via a mathematical method to indicate stress due to exposure to four different pharmaceuticals at, below and above expected environmental concentrations. The results obtained showed that even at lower concentrations than those reported for the aquatic environment, it was possible to observe a significant variation in the cellular behaviour of both algae. Thus, this study was able to demonstrate that a combination of analyses that are commonly studied separately can create a greater understanding and improve the prediction of the effects caused by micropollutants. This innovative approach can serve as a cornerstone for future studies, guiding ecotoxicological protocols and even contributing to the establishment of regulations for water quality control.

**Abstract:**

Micropollutants in aquatic resources have raised global concerns regarding the conservation of ecosystems. Although they are usually found in the environment at trace concentrations to a maximum of several µg/L, it is still necessary to address the potential risks these pollutants may represent to organisms. A multifactor analysis was conducted using two algae as bioindicators. Four different pharmaceuticals were chosen based on their occurrence in domestic wastewaters and persistency after biological treatment processes ranging from 1/8th to four-fold representative environmental concentrations over 96 h exposure. The present multifactor analysis evaluated cell size, photosynthetic capacity and growth rate. These data were later combined into a simplified single entity: “the index effect”. The results obtained showed that, even at concentrations below the environmentally relevant concentrations (ERC), the pharmaceuticals’ residues (PRs), caused a cellular behavioural variation in both organisms. In addition, the algae cultures’ response to exposure to these stressors was generally dependent on the concentration over time. By examining four different PR over three different characteristics of two types of algal bioindicators, this work covers significant and specific responses on the algae exposure cycle. This is unique research since most studies do not consider multiple parameters in the assessment of the environment risk for bioindicators.

## 1. Introduction

The release of micropollutants in aquatic resources has raised global concerns regarding the conservation of ecosystems and the need to investigate the potential consequences to species in the environment. Micropollutants can be represented by a massive and escalating range of anthropogenic or natural substances, including pharmaceuticals, personal care products, steroid hormones, industrial chemicals, pesticides and many other compounds [1]. These residues are usually found in the environment at trace concentrations, ranging from a few ng/L to several µg/L [2,3,4,5,6]. The low concentrations and the wide diversity of micropollutants not only hinders the detection and analysis procedures but also creates challenges for wastewater treatment processes [1,7].

Considering the chemical complexity and the significant variety of substances classified as micropollutants, it is relevant to focus on potentially toxic substances usually observed in the wastewater. Among these, pharmaceutical residues (PR) are becoming an emerging problem due to their constant input and persistence in aquatic resources, even in remote locations at modest concentrations levels [7,8,9]. 

In order to understand the potential toxic impacts produced by PR in the environment, two diverse cultures of algae, *Chlorella vulgaris* and *Raphidocelis subcapitata,* were selected for this study. These microorganisms were carefully chosen due to their natural abundance in the marine environment [10]; inasmuch as they demonstrate extensive application in aquatic toxicology research [7,11,12,13,14,15].

Atenolol, caffeine, carbamazepine and ibuprofen were chosen based on a recommendation list issued by a prior study, the EU PILLS (Pharmaceutical Input and Elimination from Local Point Sources) project [16,17]. Considering the PR identified by the PILLS research, tests were initially conducted to evaluate their potential toxicity.

The intention of selecting pharmaceuticals from different classes was based on the principle that each pharmaceutical would potentially have a different mechanism of action and, thus, produce a diverse ecotoxicological response. According to the literature, atenolol, carbamazepine and ibuprofen have been found in concentrations over 100 ng/L in the surface water [18]. Caffeine has been detected in the aquatic environment with concentrations closer to 6000 ng/L and at concentrations above 2000 ng/L in waste water treatment plant (WWTP) effluent [18,19].

Studies were in accordance with the Organisation for Economic Co-operation and Development (OECD) guidelines that established time and concentrations in which a chemical agent is potentially harmful to a specific biosphere; in addition, a photosynthetic study was carried out using both organisms and has addressed the algae photosynthetic state, photo acclimation and adaptation, examining the photosynthetically active radiation response [20,21].

Data obtained from the aforementioned properties were compacted into a catalogue, suggested by the present work, entitled “the index effect”. By employing multifactorial analysis, this approach enables the quantification and practical visualization of the effects in algal cultures associated with the presence of a specific PR. Furthermore, this method is able to address even limited concentrations of substances that can induce varied responses in algae according to a broad interval of exposure periods [22,23].

## 2. Materials and Methods

The evaluation procedure in this work is based on three ecotoxicity analyses. The assessment of the effects to the growth rate, cell size and photosynthesis efficiency (PE) caused by the exposure to atenolol, caffeine, carbamazepine and ibuprofen at environmentally relevant concentrations (ERC). The results are presented singularly in terms of each analysis of the inhibition and/or stimulation, and as the index effect. The index effect is the culmination of the efforts made to reach a common ground based on all previously obtained results. This last ecotoxicological approach tries to demonstrate how each previous analysis has contributed to obtain a single effect.

To compare the results of the test samples against the control samples the ordinary one-way analysis of variance (ANOVA) was used. However, to define the significance of the difference among the samples, the Tukey’s multiple comparisons test was used. The statistically significant alpha is described as *p* < 0.05 (*) and *p* < 0.01 (**), statistically highly significant as *p* < 0.001 (***) and statistically extreme significant as *p* < 0.0001 (****). All marks relate to 24 h intervals for each measurement and pharmaceutical concentration (mg/L). Results are displayed as Mean±SE, with each experiment performed in triplicate.

Both types of algae, *Chlorella vulgaris* (211/12) and *Raphidocelis subcapitata* (278/4)*,* were obtained from the Culture Collection of Algae and Protozoa (CCAP), UK. The algae were cultured in Jaworski’s Medium (JM) following CCAP protocol [24]. Synthetic wastewater was prepared following OECD guidelines [25] and all experiments were performed using a test solution containing 90% (*v/v*) of JM and 10% of wastewater (*v/v*).

To separate the stress effect caused by the investigated PR present in solution from other stressors, control samples were included. Triplicate control solutions of JM and synthetic wastewater without PR, and blank samples containing only JM, were inoculated, and incubated. The blank samples containing only JM were included to evaluate the impact of the organic load from the diluted wastewater.

The experimental concentrations used were based on the concentrations described in the literature, either measured or predicted to be found in the environment [16,18] (Table 1). These concentrations (1×) formed the baseline of the experimental setup and were complemented with concentrations several times larger (2×; 4×) or smaller (1/2×; 1/4×; 1/8×) than the baseline in order to evaluate realistic scenarios in the environment due to dilution or concentration effects in sewage works and rivers.

The LCMS/MS used for the analysis was a Thermo Scientific Q Exactive Orbitrap mass spectrometer, connected to a Dionex Ultimate 3000 RS Pump, Dionex Ultimate 3000 RS Autosampler (Temperature controlled at 10 °C) and Dionex Ultimate 3000 RS Column Compartment (Temperature controlled at 30 °C). The software used was Chromeleon^®^, Xcalibur™ and TraceFinder™. The electrospray ionisation conditions on the Orbitrap for the positive and negative polarity were sheath gas (arbitrary units) 45; auxiliary gas (arbitrary units) 10; auxiliary gas temperature and capillary temperature 300 °C. An injection volume of 10 μL was used for samples and standards. A Waters Atlantis^®^ dC18 chromatography column (150 × 2.1 mm) was employed for all assays. The mobile phase was 10 mmol ammonium formate + formic acid to pH 3.5 in water with methanol as the organic modifier. A gradient elution technique was used. Atenolol, caffeine and carbamazepine were analysed in parallel reaction monitoring mode (positive) and ibuprofen as precursor ion in negative mode. The samples were quantified against a 10-point calibration line.

LCMS/MS analysis was performed before and after the algae tests in order to determine the amount of PR that remained in the media.

Cultures were prepared in order to demonstrate an exponential growth phase, which was induced three days before the initiation of the tests in liquid culture medium. The pre-cultures were kept under the same conditions of cultivation using an incubator at 20 °C and 50% humidity. The light conditions consisted of 12 h light at 3500 lux and 12 h darkness; all samples were under agitation at 120 RPM. Algae inoculum was added, to achieve 10^6^ cells/mL, into 10 mL of test solution and so, algae were exposed to PR for a period of 96 h.

Growth capacity (cell/mL) and cell size were quantified daily by direct scanning using an automated microscope linked to the Micro Counter 1100 from Celeromics. Photosynthesis efficiency was analysed employing a dual-channel yield analyzer (ToxY-PAM; [26]).

## 3. Results

### 3.1. Cell Growth, Cell Size and Photosynthesis Efficiency

The results obtained in this study demonstrate how the three characteristics analysed in this study for *C. vulgaris* and *R. subcapitata* varied under the presence of each PR studied and were combined to form a unitary value of exposure (the index effect).

Table 2 displays the summary of the significant response obtained for all experiments for all three characteristics. It is important to state that after the statistical analysis of significance, using Tukey’s multiple comparisons test, no sample presented a *p* < 0.05 for the cell size analyses. However, given that the objective of this project was to obtain a concept that encompassed all algae characteristics, the non-significant data obtained on cell size were maintained in the index effect. It is imperative to note that while the cell size experiments were not able to statistically detect an inhibitory or stimulatory effect when analysed separately, the set of characteristics analysed together, which includes this specific feature, can discern the stress intensity regarding cell size in the total or universal result.

### 3.2. Index Effect

The index effect data are presented as normalised or absolute results. The results on the y axis show inhibition yielding a positive percentage value and stimulation effects producing a negative value. All PR exposure results were normalised in comparison with the control, drug free values. Outcomes were normalised against the control sample to correct for the organic load in the synthetic wastewater, which can serve as a nutrient source for the bioindicator organisms. This means that only the influence of the pharmaceutical upon the bioindicator culture was being examined.

**Figure 1:**
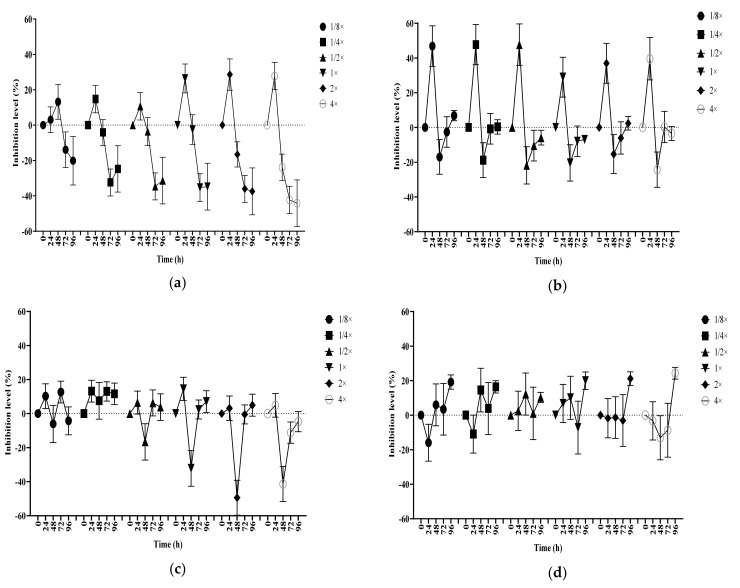
*C. vulgaris* index effect by the exposure to 4 different pharmaceuticals. (**a**) Atenolol; (**b**) Caffeine; (**c**) Carbamazepine; (**d**) Ibuprofen. See Table 1 for explanation of drug concentration linked to the sample number.

**Figure 2:**
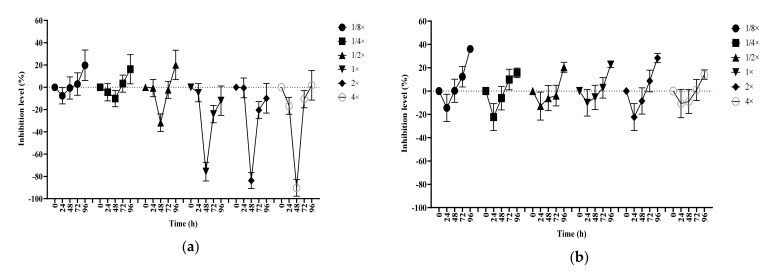

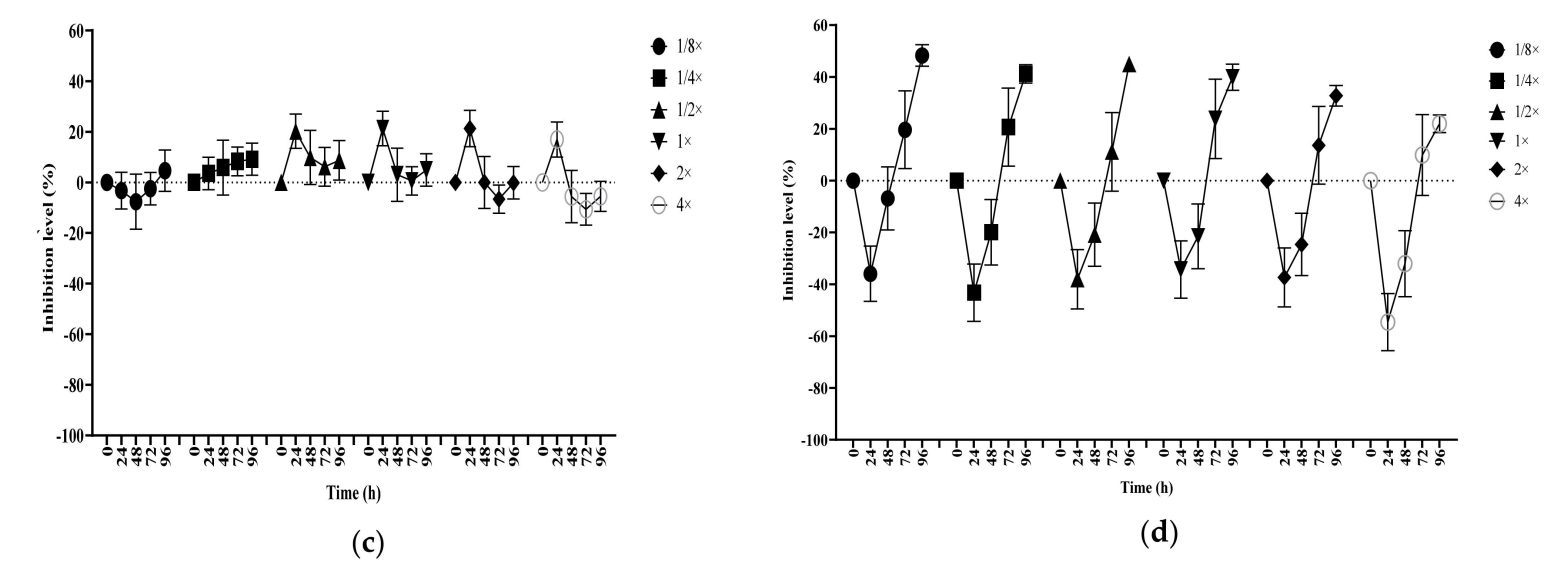
*R. subcapitata* index effect by the exposure to 4 different pharmaceuticals. (**a**) Atenolol; (**b**) Caffeine; (**c**) Carbamazepine; (**d**) Ibuprofen. * See Table 1 for explanation of drug concentration linked to the sample number.

Data obtained for *C. vulgaris* and *R. subcapitata* following exposure to atenolol, caffeine, carbamazepine and ibuprofen can be found in the following graphs combined as the index effect (Figure 1 and Figure 2).

## 4. Discussion

From the four PR tested in this study only carbamazepine presented a solo effect on *C. Vulgaris’* growth and photosynthesis efficiency. Both analyses presented a stimulatory effect caused by the presence of carbamazepine.

In the case of the other three stressors present in the test samples at or around ERC, it was observed that there was a general inverse relation between stimulation and inhibition caused on the algae growth and on photosynthesis efficiency. These results are aligned with the literature where some PR can inhibit the growth rate of algae by affecting the growth or photosynthesis process directly [27,28,29], while some pharmaceuticals, at low concentrations (<10 mg/L), can cause positive effects on both the structure and function of algal cultures, algal growth, chlorophyll and lipid accumulation [30]. Thus, there is a requirement to take both the growth and the photosynthesis process in consideration when assessing the ecological risk of a toxicant.

The *C. vulgaris* index effect (CIE) obtained following atenolol exposure demonstrated visually how the increase in the pharmaceutical concentration intensified the inhibitory and/or stimulatory effects on this alga (Figure 1a). Atenolol’s CIE presented a significant inhibitory trend from concentrations equal or higher than the ERC in the first 24 h. Followed by a stimulatory downward curve, the index effect reached a significant stimulus peak at 72 h. The curve elongation followed increases in atenolol concentration, showing a direct dependence of the algae index effect on the concentrations tested at that time. In the same manner, the exposure of *R. subcapitata* to atenolol presented a concentration dependent index effect (Figure 2a) but with a different behaviour when compared to that observed for CIE. The *R. subcapitata* index effect (RIE) was observed to show a strong stimulation response to the atenolol with a significant peak at 48 h and a possible afterward inhibition at 72 h. To the same degree that was observed on the CIE, the curve variation presented a dose dependent inhibition/stimulus variation to concentrations equal or higher than the ERC.

Caffeine’s CIE (Figure 1b) and RIE (Figure 2b) indicated different responses to the presence of caffeine at all concentrations tested. While its effect on CIE presented a significant variation up to 48 h, reaching levels between 50% and −20% on the inhibition scale, a significant variation in the RIE was only observed after 96 h of the experiment with inhibition peaks at 20% for most of the samples and an accentuated 35% index effect inhibition for sample 1. Unlike atenolol observations, the index effect obtained for caffeine did not show any concentration dependence. It was possible to observe that the 24 h inhibition effect in samples with lower than ERC levels were more pronounced for CIE when compared to the other samples, and, with RIE, all samples presented the same pattern with no great difference from sample 1 to sample 6.

When exposed to carbamazepine, the CIE indicated a trend in stimulation at 48 h and a slight inhibition at 72 h (Figure 1c); for RIE, an opposite response was noted with initial inhibition at 24 h and stimulation at 48 h (Figure 2c). However, both organisms were significantly affected at the same level of concentration during those periods. The exposure of algae to the carbamazepine caused the overall RIE to show up to 20% stimulation in these samples. Although it was not possible to note any concentration relationship between carbamazepine and CIE, the samples with ERC and above presented a correlation between the pharmaceutical concentrations and an approximate 40% stimulation of the index effect.

Finally, the CIE observed in the presence of ibuprofen showed a 20% average inhibition over all combinations of ERC tested after 96 h exposure (Figure 1d). Even though there was not an observable significant difference in the data obtained prior to the last day of the experiment, some similarities were observed between the curves obtained for the different concentrations at different periods of time. The results obtained for ibuprofen’s RIE (Figure 2d) were much more consistent when compared to those obtained for CIE. The impact of the PR on *R. subcapitata* culture were even clearer. In the first 24 h, all samples showed an index effect value 40% higher than the control. After this initial stimulus, it was possible to observe the transition and the increase in the value inhibition on the data obtained at 48 h and 72 h. Finally, on the last day of analysis, all test samples showed values 20% to 40% smaller than the control, with strong significant difference, all *p*-values were smaller than 0.0001.

Therefore, it can be stated that at some points, even at concentrations below the ERC, the PRs that were tested caused a cellular behavioural variation in both organisms. Moreover, the algae cultures’ response to exposure to these stressors was generally dependent on the concentration over time.

## 5. Conclusions

A broad ecotoxicological concept (index effect) for the description of potential harmful effects on the environment was developed and evaluated for the effect of atenolol, caffeine, carbamazepine and ibuprofen on *C. vulgaris* and *R. subcapitata*. This index effect combined observations on cellular size, reproductive capacity and photosynthetic efficiency, and demonstrated that a combination of analyses that are commonly studied separately can create a greater understanding and prediction of the combined ecotoxicological effect. The results found in this study endorse the view that a multifactorial approach, such as the index effect, should be considered when investigating the exposure of algae to pharmaceuticals.

The multifactorial method allowed an understanding of the moment (in hours) when the organism is facing a critical point that may impair its function.

In conclusion, this study shows that the current models applied for ecotoxicity assessment underestimate the overall impact of a compound when only one feature of the organisms define the pollutant level of toxicity. The application of a more complete method, such as the index effect, allows the monitoring of the potential toxicity of pharmaceuticals that do not just consider population reduction as the sole risk factor. It also suggests that the multifactorial, ecotoxicological approach supports the characterisation of the combined toxicity in the aquatic environment while demonstrating the sensitivity of bioindicator species following their exposure to a range of PR at ERC. Finally, the index effect allows the evaluation of the bioindicators’ function under multiple scenarios including those at environmental concentrations.

Along with the results obtained for the index effect, the pharmaceutical bioaccumulation potential may represent a risk to other organisms based on the trophic web. Moreover, the PR-induced enhancement of algae development observed in this study, can be associated with the occurrence of imbalances in the aquatic ecosystem, as an organism can overgrow substantially and, in critical cases, impair other species’ survival.

## Figures and Tables

**Table 1:** Environmentally relevant pharmaceutical concentrations and scenario testing ranges.

Pharmaceutical	Concentrations [μg/L] at Different Scenarios					
	1/8×	1/4×	1/2×	1×	2×	4×
Atenolol	2.94	5.88	11.75	23.50	47.00	94.00
Caffeine	9.13	18.25	36.50	73.00	146.00	292.00
Carbamazepine	2.09	4.19	8.38	16.75	33.50	67.00
Ibuprofen	2.00	4.00	8.00	16.00	32.00	64.00

**Table 2:** Pharmaceutical concentrations and their effect on *C. vulgaris* and *R. subcapitata* cultures.

PR	ERC (μg/L)	Species	Critical Effect (Average Observed)	Concentration Range of Observed Effect (μg/L)
**Atenolol**	23.5	*C. V*	50% Growth Stimulation at 96 h	2.94–94
			5% Photosynthesis inhibition at 96 h	2.94–94
		*R. S*	70% Growth stimulation at 48 h	2.94–94
			5% Photosynthesis inhibition at 96 h	2.94–23.5
**Caffeine**	73	*C. V*	25% Growth inhibition at 24 h	9.13–292
			5% Photosynthesis stimulus at 48 h	36.5–146
		*R. S*	15% Growth inhibition at 96 h	9.13–292
			10% Photosynthesis stimulation at 72 h	292
**Carbamazepine**	16.75	*C. V*	40% Growth stimulation at 48 h	16.75–67
			5% Photosynthesis stimulation at 72 h	67
		*R. S*	Growth effect	Non-Detected
			3% Photosynthesis inhibition at 24 h	33.5–67
			5% Photosynthesis inhibition at 72 h	67
**Ibuprofen**	16	*C. V*	20% Growth inhibition at 96 h	16–64
			Photosynthesis Efficiency	Non-Detected
			40% Growth stimulation at 24 h	4–64
		*R. S*	40% Growth stimulation at 48 h	64
			20% Photosynthesis inhibition at 72 h	4–16

*C. V: Chlorella vulgaris, R. S: Raphidocelis subcapitata*.

## Data Availability

The data presented in this study are available on request from the corresponding author.
